# Lysozyme crystallization in hydrogel media under ultrasound irradiation

**DOI:** 10.1016/j.ultsonch.2022.106096

**Published:** 2022-07-18

**Authors:** Mariia Savchenko, Manuel Hurtado, Modesto T. Lopez-Lopez, Guillermo Rus, Luis Álvarez de Cienfuegos, Juan Melchor, José A. Gavira

**Affiliations:** a Universidad de Granada (UGR), Departamento de Química Orgánica, Unidad de Excelencia Química Aplicada a Biomedicina y Medioambiente (UEQ), C. U. Fuentenueva, Avda. Severo Ochoa s/n, E-18071 Granada, Spain; b Universidad de Granada (UGR), Departamento de Estadística e Investigación Operativa, Spain; c Universidad de Granada (UGR), Departamento de Física Aplicada, C. U. Fuentenueva, Avda. Severo Ochoa s/n, E-18071 Granada, Spain; d Laboratorio de Estudios Cristalográficos, Instituto Andaluz de Ciencias de la Tierra (Consejo Superior de Investigaciones Científicas-UGR), UEQ, Avenida de las Palmeras 4, 18100 Armilla, Granada, Spain; e Departamento de Mecánica de Estructuras e Ingeniería Hidráulica, Ultrasonics Lab TEP-959, Universidad de Granada, Spain; f Unidad de Excelencia Modeling Nature MNAT, Universidad de Granada, Spain; g Instituto de Investigación Biosanitaria Ibs, GRANADA, Granada, Spain

**Keywords:** Protein crystallization, Nucleation, Ultrasound, Hydrogels, Lysozyme

## Abstract

Sonocrystallization implies the application of ultrasound radiation to control the nucleation and crystal growth depending on the actuation time and intensity. Its application allows to induce nucleation at lower supersaturations than required under standard conditions. Although extended in inorganic and organic crystallization, it has been scarcely explored in protein crystallization. Now, that industrial protein crystallization is gaining momentum, the interest on new ways to control protein nucleation and crystal growth is advancing. In this work we present the development of a novel ultrasound bioreactor to study its influence on protein crystallization in agarose gel. Gel media minimize convention currents and sedimentation, favoring a more homogeneous and stable conditions to study the effect of an externally generated low energy ultrasonic irradiation on protein crystallization avoiding other undesired effects such as temperature increase, introduction of surfaces which induce nucleation, destructive cavitation phenomena, etc. In-depth statistical analysis of the results has shown that the impact of ultrasound in gel media on crystal size populations are statistically significant and reproducible.

## Highlights

• A non-invasive low energy ultrasound bioreactor for proteins sonocrystallization.
• Agarose minimize convention and sedimentation facilitating result interpretation.
• Ultrasound irradiation induce lysozyme nucleation in solution and in gelled media.
• Irradiated samples produced more and homogeneous crystal size distribution.

## 1 Introduction

Protein crystallization is an essential tool to decipher the three-dimensional structure of a protein in its crystalline state by means of X-ray diffraction, as well as the catalytic center of an enzyme, allowing us to understand its mechanism of action [1]. Therefore, protein crystallization is needed, among others, in fields of structural biology and drug development and discovery. Moreover, protein crystallization can also be useful in protein purification and isolation being used in biopharmaceutical industries routinely [2], [3]. Nevertheless, due to the high molecular weights and dynamic nature of proteins in solution; protein crystallization is a complex process that requires high supersaturation ratios and usually proceeds by slow nucleation and crystal growth kinetics [4], [5]. In this regard, it is fundamental to develop novel techniques or protocols that allow a more efficient and reliable crystallization process. Protein crystallization in gel media has turned out to be an excellent strategy to obtain protein crystals of high quality and size, ideal for X-ray diffraction, mainly due to the reduction of convection currents, avoiding crystals sedimentation and temperature or concentration gradients [6]. At such, crystallization in gels is similar to crystallization under reduced gravity condition producing crystals of excellent quality [7]. Moreover, gel fibers get incorporated within the crystals transforming them into new composite materials [8], [9].

Protein crystallization is also highly affected by external stimuli, such as electric [10], [11], [12], [13], [14] and magnetic fields [15], [16], [17], [18], light [19], [20], audible sounds [21], microwave [22] and ultrasound (US) irradiation [23], [24], [25], [26], having a direct influence on nucleation [14]. Among these strategies, in the last decade, sonocrystallization, that is, the application of US in crystallization process, is receiving a growing interest since it has shown to: increase nucleation rate, reduce the induction time and the metastable zone width, increased crystal growth rate, reduced agglomeration, and improve crystal quality and size distribution [27], [28], [29], [30]. The effect of ultrasounds on the kinetic of crystallization has been known from more than 80 years with an early review already published in 1967 and in which the multiple possible mechanisms i.e. cavitation, agitation, cooling effect or mechanical vibration were hypothesized [31]. Besides an extended number of its effects on the nucleation and growth of inorganic/organic crystals and even fat [32], only recently the effect over protein crystallogenesis has been reported [25], [26]. Nevertheless, the application of US in protein crystallization has not been systematically studied and sonication factors such as, source, power, time, direction and amplitude of the ultrasound waves, are still to be investigated. Moreover, any study carries out under standard crystallization set-ups will be affected by sedimentation and convection mass transport hindering the interpretation of the results. Only recently, Ferreira and co-workers have explored the effect of US pulse over lysozyme microdroplets [25]. In order to extend the potential use of sonocrystallization to bigger volumes and to explore the feasibility of this technique in combination with other crystallization protocols relevant for industrial crystallization, we have chosen to include agarose to minimize convection and crystal sedimentation.

Agarose is one of the most used media to gel a crystallizing solution of inorganic and organic compounds [33], coordination polymers [34] or proteins [35], to produce new polymorphs [36] or to emulate *in vivo* media in biomineralization studies [37]. Agarose gels are composed of interconnected uncharged linear polysaccharide chains easily obtained by cooling the sol below its gelling temperature and classified as physical gel since the interactions between polysaccharide chains are non-covalent. It uses is widely spread in biochemistry labs as supporting material for horizontal electrophoresis. The physical properties of the gel have been well characterized and it is well known that below the critical gel concentration of 0.12 % (w/v) agarose solutions behave like non-Newtonian fluids, while above this concentration it behaves as a regular viscoelastic gel [38]. Moreover, at a concentration as low as 0.04 % (w/v) agarose gels are able to overcome buoyancy and crystal sedimentation [39]. Also, agarose gels have been previously used to study the influence of a magnetic field while avoiding sedimentation and the associate effects of convective mass transport on nucleation [18], [40].

In the present work, we have studied the influence of US in protein crystallization at different agarose concentration. To carry out this study a novel US bioreactor has been specifically designed to have a more precise control over the wave parameters of irradiation. In-depth multivariate statistical analysis have shown that, as already published, US induced the nucleation of lysozyme over the inductor effect of agarose [41], [42]. Our results also shown that above a threshold concentration of agarose the US effect is deflected probably due to the damping effect of agarose fibers. The results obtained by this set-up are statistically significant allowing us to stablished a clear effect of the ultrasound on the nucleation of lysozyme under studied conditions.

## 2 Materials and methods

### 2.1 Reagents and materials

Lysozyme (62971, HEWL, three-times crystallized powder) and sodium acetate (AcONa 99 %) were purchased from Sigma-Aldrich (Madrid, Spain). Lysozyme was dissolved in 50 mM AcONa, dialyzed (24 h) against 50 mM AcONa (pH 4.5) in a ratio 1:1000 at 4 °C and concentrated by centrifugation at 4 °C (g = *5000/25 min) using 10-kDa cutoff Centricon concentrators (Amicon) to ≈ 150 mg mL^−1^ determined spectrophotometrically at 280 nm using a theoretical value for the extinction coefficient of 2.56 mL mg^−1^. Then the solution was filtered through a 0.45 μm pore-size filter membrane system (Millipore). Sodium chloride (Sigma-Aldrich, Madrid, Spain) was prepared at 20 % (w/v) in 50 mM AcONa (pH 4.5) and used as stock solution. Solutions of NaCl at desired concentration were prepared by diluting with 50 mM AcONa and filtered through a 0.45 μm pore-size filter membrane system (Millipore) prior using it.

Agarose D5 with a melting point of 92 °C and gelling point of 37 °C was supplied by Hispanagar (Madrid, Spain). Agarose sols with desirable concentration were obtained by dissolving agarose in 50 mM AcONa (pH 4.5) and heated at 90 °C to get a homogeneous transparent solution. Then the solution was cooled down to 50 °C and kept at this temperature until finally mixed with the protein and precipitant solution.

### 2.2 Crystallization experiments

**Fig. 1:**
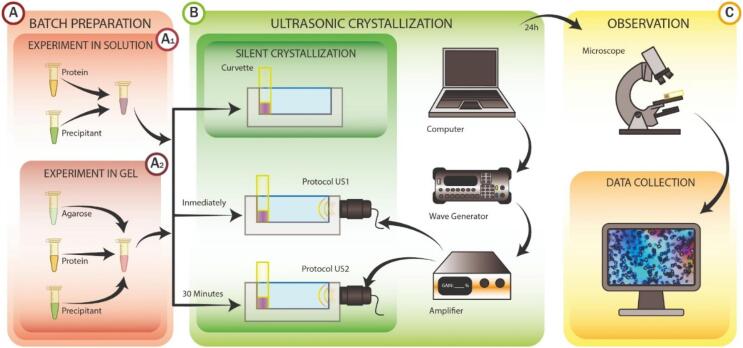
The experimental scheme: A) Preparation of the samples A1: Experiments in solution, A2: Experiments in gels made with agarose; B) US-Bioreactor design and sonification procedure and illustration of the set-up; C) Observation of the samples and data analysis.

Batch method was selected to study the influence of ultrasonic waves on lysozyme crystallization in solution (Fig. 1.A). For the experiments in solution (Fig. 1.A1), lysozyme, NaCl and AcONa were mixed together in one Eppendorf tube, homogenized and divided in three aliquots of 100 µL using micro spectrophotometer visible-cuvettes (Brand, GMBH, CO-KG, Germany) and kept at 20 °C.

To select the appropriate conditions, we screened lysozyme (25–50 mg mL^−1^) and NaCl (3.5–5 % w/v). We have selected the final concentration of lysozyme 40 mg mL^−1^ and NaCl 4.3 % (w/v) for the experiments in un-gelled solution and 40 mg mL^−1^ and NaCl 4.0 % (w/v) for the experiment in agarose gels. The evolution of the experiments was followed by standard optical microscopy (Nikon AZ100 zoom 2x2x0.6) observing the formation of tiny crystals in all the samples (un-gelled solution as well as in samples with agarose gels) after 1 h.

The influence of agarose (Fig. 1A2) was studied by varying the final amount of agarose in the system: 0.010 %, 0.025 %, 0.050 %, 0.100 % and 0.200 % (w/v). We set the upper limit at 0.200 % (w/v) to cross the critical concentration value of 0.120 % w/v defining the transition of the viscoelastic behavior [43]. For each experiment three aliquots were prepared and divided as “silent” for the sample without ultrasonic influence, “Protocol US1” for the aliquot immediately irradiated for 30 min long and “Protocol US2” for the aliquot exposed to the ultrasonic influence 30 min after preparation and irradiated for 30 min (Fig. 1B). All the experiments were performed threefold for statistical significance.

Number and size of crystals were evaluated after 24 h by optical microscopy using the Image-Focus-Alpha software of the Nikon AZ100 microscope (zoom 2x2x0.6). Crystals number were counted manually and size measured along the c axes from crystals clearly identified. Each image was divided in 25 equal regions (5 columns × 5 rows) avoiding zones near cuvettes borders where it was not possible to see the crystals clearly, and all the crystals were counted in all the regions. Crystals size were analyzed by measuring a minimum of 100 crystals for each replicate thus, a minimum of 300 measurements were analyzed for each condition. Taking into account all the controls and different experimental conditions a total of 5400 measurements were done.

### 2.3 US-Bioreactor design

**Fig. 2:**
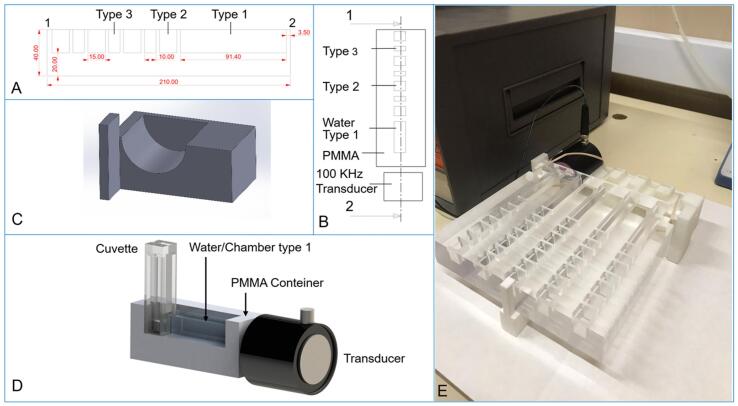
A and B) Shows the lateral and top 1–2 transversal section views, respectively. The chamber Type 1 was designed to hold the cuvette and it is located at 3.5 mm in front of the block. The dimensions of this chamber had 15 mm width, 12 mm of effective high and 91.4 mm of thickness (approximated volume of 16.4 cm^3^). The chambers Type 2 and Type 3 were introduced to hold several cuvettes in the future considering different pressures changing the main configuration. C) Exhibits the 3D printed support to maintain the ultrasonic transducer (100 KHz) in a correct position to ensure the alignment of the propagating waves. D) 3D schematic full configuration of the US-Bioreactor. E) Picture of the US-Bioreactor prepared for the experimental irradiation of gels.

To avoid a direct contact of the US emitter with the gel/crystallization media that could cause gel disruption or serve as heterogeneous nucleant, a US-bioreactor was designed, manufactured and prototyped in the Ultrasonics lab at the University of Granada. It consists of a container to hold the cuvette, a polymethylmethacrylate (PMMA) chamber that was chosen due to its mechanical and low-density properties and ultrasonic transducers (Fig. 2). In parallel, the propagation of the waves was allowed and maximized without loss of amplitude because of impedance between mediums.

The support of the transducer designed in Fig. 2C has the function of holding the transducers, to be stable and perpendicular to the block with a specific geometry showed in Fig. 2A-B. The decision was focused at allowing the whole wave incidence into the chamber of the samples where the cuvette is integrated, to ensure maximum irradiation. A pressure on the block is also required to have a direct transmission avoiding possible air bubbles. Moreover, the bottom of the cuvette must coincide with the central axis of the transducer to achieve optimum intensity due to the maximization of the resolution of beam in the central axis. The propagation front of the mechanical ultrasonic waves is transmitted perpendicularly to the surface of the transducer in contact with the container.

The relationship between wave propagation, acoustic impedance of the materials, near field, the possible temperature rise of the transducers and mechanical nature of the sample have been also considered for the design of the container and configuration of the ultrasonic transducers. The impedance is characterized as a product of density and velocity of sound of the material as follows:

(1) Z=ρ∙c

where (Z) is the impedance, (ρ) is the density and (c) is the velocity of the wave through material.

The mechanical parameters of the US-bioreactor materials were characterized to analyze the propagation through the sample, the impedances of PMMA, water and the cuvette-polystyrene (PS) were also considered as is shown in Table 1 according to Equation (1), (2). Table 1 describes densities, velocities of sound and impedances of these materials.

**Table 1:** Characterization of PMMA, Water and PS in terms of impedance to quantify the effect of the ultrasonic propagation in the sample.

Material	(ρ) Density Kg/m^3^	(c) Velocity (m/s)	(Z) Impedance (Pa.s/m)
PMMA	1180	2765	3,263,183
Water (27°)	993	1525	1,515,584
Polystyrene (PS)	612	2340	1,432,080

The transmission coefficient at the different interfaces is also necessary to determine the pressure in the sample after propagation through the different layers. It is explicitly formulated as:

(2) $$D=\frac{{4Z}_{1}Z_{2}}{{\left[Z_{1}+Z_{2}\right]}^{2}}$$

where (D) is the transmission coefficient, (Z_1_) is the impedance of material 1 and (Z_2_) is the impedance of material 2. The calculated values are summarized in Table 2.

**Table 2:** Transmission coefficients between water, PMMA and PS.

D	PMMA	Water/gel	PS
PMMA	1	0,86	–
Water/gel	0,86	1	0,992
PS	–	0,9992	1

Transmitted pressure has been also derived for the transition between two media according to equation (2) (Fig. 2B). While, the wave is oscillating at 100 kHz of frequency from the ultrasonic transducer, it travels through the PMMA, after the water and finally the polystyrene until get in contact with the gel or solution sample. For this calculation, we have approximated the density and the velocity of sound of the gel or solution equal to water, because they have almost the same value. Thus, acoustic pressure in water has been quantified as 618 Pa, considered well below the cavitation limit at this frequency, and theoretical derivation of acoustic pressure inside the gel or solution has been extracted according its transmission coefficient D,

From water to polystyrene → P = 618 Pa ⋅ 0,99 = 611,82 Pa

From polystyrene to gel or solution → P = 611,82 Pa ⋅ 0,99 = 605,7 Pa

Several reports in the literature show that the response to US is triggered by temperature actuation [44], [45]. Therefore, to verify this point, temperature is also measured in two intervals, at 5 min and at 15 min. The temperature actuation is considered fast, so it is not necessary to measure it over a longer period of time. We have used a classical thermometer to control this effect, whose accuracy is 0.01 °C. For each case, in 100 kHz of frequency, the measurements on nearest chambers to ultrasonic transducer are lower than <0.01 °C, i.e., the heating effect is negligible with our configuration.

Otherwise, the samples were placed in the cuvette (Fig. 2D) to enhance the wave propagation. So that the chosen distance was approximately 8 cm from the transducers to avoid the near field area (in this region the sound pressure levels vary considerably in terms random positions of energy and it is difficult to control the sound pressure homogeneity), the threshold distance is calculated according to Equation (3) as follows,

(3) $$N=\frac{D^{2}f}{4c}$$

where (N) is the near field, (D) is the diameter, (f) is the frequency and (c) is the velocity of sound of the incident the wave that characterize the media of propagation (Table 3).

**Table 3:** Near field calculation in terms of frequency, velocity and diameter of the transducer.

(f) Frequency (kHz)	(D) Diameter (cm)	(c) Velocity (m/s)	(N) Near field (cm)
100	4,5	1525	3,32

In this research, vaseline was used as coupling gel due to the duration properties of this set of experiments, the common coupling gels are dried in a few hours. Additionally, conventional rubbers bands have been added to the US-bioreactor support to fix the transducer to ensure an optimal pressure of contact and no displacement during the irradiation experiments.

### 2.4 Experimental US configuration

The ultrasonic set-up of the experiment was designed to analyze the differences between the effect on crystallization of the solution and agarose gels at different concentrations (Fig. 1). In consequence of the limitation of the wave generator to 10 V, an amplifier has been needed to reach 180 peak-to-peak V as optimum scale level to generated the desired effect. The waveform used was configured with a 5 % duty cycle and 50 ms of burst period simulating a continuous propagation of the wave. The amplified wave signal was emitted at 100 kHz of central frequency according to a compatible wavelength with the sample dimensions [23].

**Fig. 3:**
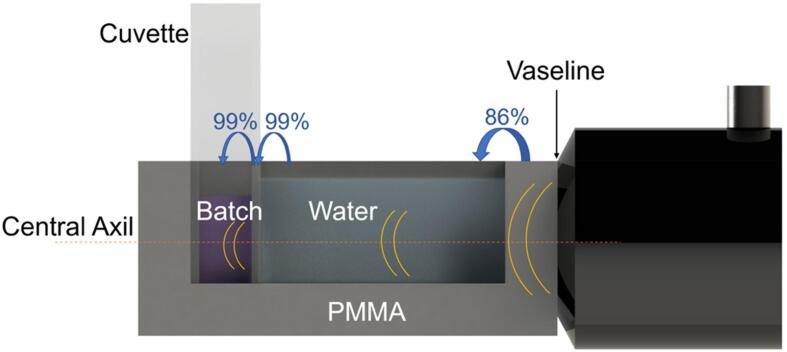
3D-CAD illustration of the prototyped of US-Bioreactor with a recreation of multilayer media propagation through vaseline, PMMA, water, polystyrene and gel sample.

For this design requirements, contact transducers have been selected due to their properties as non-tuned devices. They provide a damped broadband that minimizes the undesired noise. This type of transducers are usually adequate in Non-Destructive Testing (NDT) applications and biomedical engineering [46]. Then, the reason to choose piezo-electric transducers is that they are made of a single PZT ceramic element. They typically generate a longitudinal monochromatic wave in contact with the sample, therefore, they meet the specifications to generate a monochromatic wave to be propagated in multilayer media (Fig. 3).

### 2.5 Rheological characterization of hydrogels

For the rheological characterization we used a Bohlin CS10 controlled stress rheometer, provided with a measuring geometry of concentric cylinders with grooved surfaces.

(a) Kinetics of gelation

We measured the gelation kinetics of the agarose solutions used for the crystallization during 1 h (that includes the time applied for both Protocol US1 and Protocol US2) by subjecting them to oscillatory strain of 1 Hz of frequency and 1 Pa of the stress. The measurements started 10 s after the mixture of the components (the delay resulted from the time required to lower the inner cylinder to the measuring position and to start the measurement). Three different samples were measured to ensure statistical significance of the results. The mean values and standard deviations of each magnitude were provided in this work.

(b) Mechanical properties

We characterized the mechanical properties of 0.100 % and 0.200 % (w/v) agarose gels before and after the irradiation of US (using both Protocol US1 and US2). For this aim, we obtained the storage (G’) and loss (G’’) moduli of the gels as functions of frequency (from 0.1 to 10 Hz) at a constant stress of 1 Pa. Three different samples were measured to ensure statistical significance of the results. The mean values and standard deviations of each magnitude were provided in this work.

### 2.6 Statistical analysis

To explore an inference about the differences between the samples with and without US at two levels of exposition, a multiple regression analysis via ANOVA was considered. The first step was to check the proof of normality *via* the Kolmogorov-Smirnov and Shapiro-Wilk tests. They were calculated to determine the normality of the size and number of observed crystals, respectively. In the cases where the distribution of the variable was non-normal, Kruskal-Wallis test was performed to obtain the simultaneous mean differences by experiment and group of treatment or level (Control, US1 and US2). Furthermore, they have been corroborated with Dunn’s test to assess the p-value significance intra-group. For the parametric distributed variables, we have applied classical one-way ANOVA statistical methodology to describe the differences of means by group of treatment.

The p-values have been compared with the significance level to evaluate the null hypothesis where there were no differences between means or when the null hypothesis indicates that the population means are all equal. A significance level type I error of 0,05 was considered to be the minimum accepted level that denotes a difference between means. The notation that we have included hereafter is * p < 0,05, ** p < 0,001 and *** p < 0,0001 when the differences between means are statistically significant. For the record the individual analysis of each experiment in number and crystals size are included in the supplementary material Figures S1 and S2.

## 3 Results and discussion

Nucleation is a stochastic phenomenon that requires to overcome an energy barrier. This energy barrier can be reduced by increasing the supersaturation but, on the other hand, an excess of supersaturation reduces the ability to control the nucleation time and density [4]. There are different ways to overcome the longest induction time imposed by the thickness of the metastable zone. To slim down the metastable zone, nucleation has been promoted by using different surfaces ([47] and references here in), gels [41][42], and external fields such as light irradiation, electric and magnetic field or US ([14] and references here in), all of them promoting the nucleation and therefore reducing the nucleation induction time and increasing the number of crystals [14].

The ultrasonic activation of metastable solutions to induce nucleation is the standard application of sononucleation technique to control crystal size distribution, morphology or polymorph selection [[48], [49]]. In order to study the influence of US at a fixed supersaturation with an uncertainly metastable zone, the application of US at different times, after the setting up of the crystallization experiments, seems to be the simple way.

We proposed a set-up in which US is generated externally to the bulk crystallization solution and which includes a hydrogel media to avoid crystals sedimentation and convection so that the US effect could be un-couple from any mass transport effects. To test the efficiency of our set-up we carried out a first set of experiments in an un-gelled solution. We tested a range of supersaturation values by changing the lysozyme concentration, from 25 to 50 mg·mL^−1^, NaCl concentration from 3.5 % to 5.0 % (w/v) and the irradiation time set at 10 s, 10 min and 30 min. From this initial screening we determined that using 40 mg·mL^−1^ of lysozyme and 4.3 % (w/v) of NaCl the nucleation induction time moved in the range of the initial 60 min and therefore fitted our experimental requirements. We also determined that short irradiation periods, 10 s or 10 min, after preparation did not influence the nucleation behavior and therefore a minimum of 30 min of irradiation was required which could be located within the 60 min of induction time, i.e. at the beginning (Protocol US1) and 30 min after the preparation (Protocol US2). Number and size of crystals were evaluated after 24 h by optical microscopy using the Image-Focus-Alpha software of the Nikon AZ100 microscope (zoom 2×2×0.6).

**Fig. 4:**
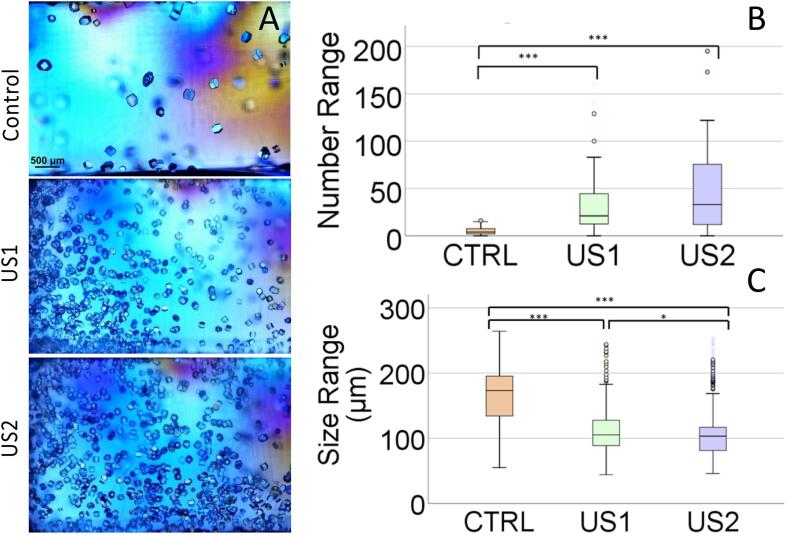
Row A) Shows lysozyme crystals obtained in solution under silent condition (Control) and ultrasonic irradiation for 30 min immediately after preparation of the experiment (US1) and 30 min after preparation (US2). The statistical analysis of the number (B) and size (C) of the crystals are shown for the three replicates. The scale bar in the optical microscopy images is 500 µm in all the pictures.

As expected from previous results under similar conditions [26], the irradiated sample in solution showed an increase of the number of crystals, of smaller sizes, independently of the used protocol (Fig. 4 and Figure S3). Without irradiation, 50 % of measured crystals were comprised between 150 and 200 µm while near 70 % of the immediately irradiated crystals (US1) showed sizes ranging 50–100 µm and when irradiated 30 min after sample preparation (US2) almost 90 % of the crystals showed sizes ranging 50–100 µm. Notably this novel bioreactor seems to be ideal to exert a control over the nucleation process. Moreover, the higher control over the crystal size observed in protocol US2 could be explained as following: i) since US2 is applied later, crystalline material is already formed; ii) the application of the US over this already formed nuclei may or could disaggregate the crystalline material and iii) the disaggregated fragments have now the opportunity to grow giving rise to a narrower crystal size distribution.

To avoid crystal sedimentation, agarose, at different concentrations, was used as a media. Agarose is a well-known nucleation induction media fully characterized under regular condition but not under the influence of US. Therefore, firstly, we fully characterized the influence of our selected protocol over the gel/gelation behavior of agarose.

In order to investigate how the ultrasound may affect the kinetic of gel formation and the mechanical properties of the gel we stick to the concentration range in which agarose transit from non-Newtonian fluid (0.100 % w/v) to a regular gel (0.200 % w/v) [38].

**Fig. 5:**
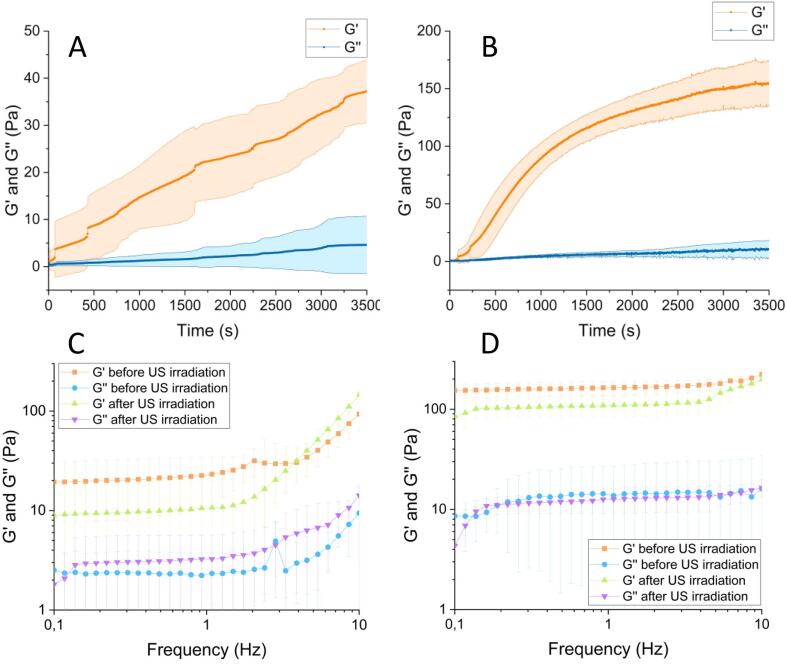
Gelation kinetic of 0.100 % (A) and 0.200 % (B) solutions of agarose. Darker lines represent the mean values, whereas the lighter bands around them represent the standard deviations. The bottom part showing the viscoelastic moduli as a function of frequency at a constant stress of 1 Pa for 0.100 % (C) and 0.200 % (D) agarose gels before and after applying the protocol US1. Darker lines represent the mean values, whereas the lighter bands around them represent the standard deviations. Note that the nonsymmetric appearance of standard deviations is due to the logarithmic scale.

As expected, at agarose concentration below 0.100 %, no gel behavior was obtained i.e. storage modulus (Ǵ) being always smaller than loss modulus (G′′), even after monitoring for 1 h. For 0.100 % and 0.200 % (w/v) agarose concentration G′ was higher than G′′ from the very beginning of the measurements. G′ > G′′ is typical of gel-like samples and therefore, it can be concluded that gel point was reached very quickly after the solutions were prepared. Nevertheless, as observed in Fig. 5A-B, G′ increased over time, which indicates that gelation proceeded after the gel point was reached, and no steady state was reached after 3500 s for 0.100 %. For 0.200 % the initial enhancement of G′ was very abrupt, but after 2500 s a trend towards a steady state was observed indicating the gel formation. Fig. 5C-D, shows the viscoelastic moduli as a function of frequency for a constant shear stress of 1 Pa after the gelation was completed. These curves seem to corroborate that both samples containing 0.100 % and 0.200 % of agarose demonstrated a gel-like behavior, however, values of G′′/G′ are in the range 0.1–1, mostly for sample 0.100 %, typical of weak gels [50]. Although at first it looks like there is some differences on the viscoelastic moduli between irradiated and non-irradiated samples, the standard deviation clearly overlaps indicating that there are not significant differences.

Also note that in the double logarithmic scale, G′ and G′′ are weakly dependent on frequency, which is typical of gels. We also investigated if the application of US2 could affect the gel behavior. As it is illustrated in the supplement material (Figure S4) we did not observe any effect.

Therefore, from our results it could be concluded that both samples are gels, with sample 0.100 % agarose presenting weaker mechanical properties than 0.200 % agarose, which are not affected by the US irradiation.

**Fig. 6:**
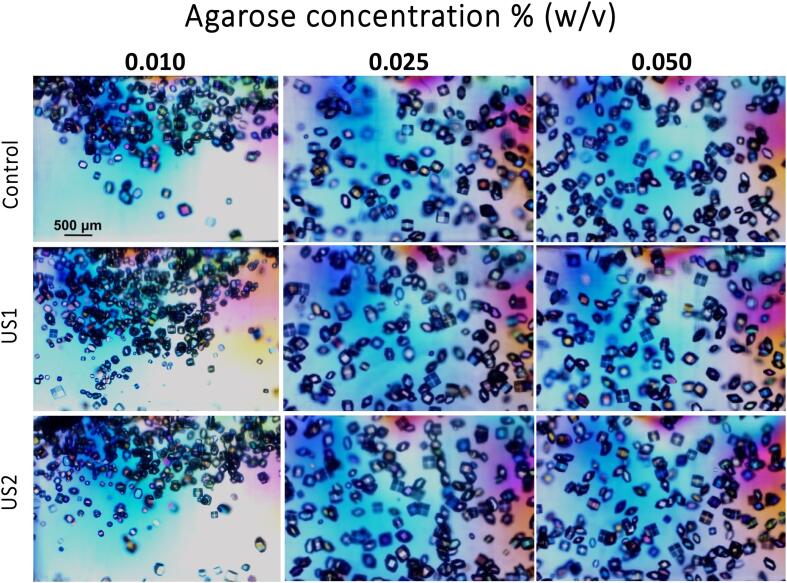
Lysozyme crystals obtained in solution under silent condition (Control) and ultrasonic irradiation for 30 min immediately after preparing the experiment (US1) and 30 min after preparation, irradiated for 30 min (US2). From left to right it is shown the result in solution and increasing concentration of agarose. The scale bar in the optical microscopy images is 500 µm in all the pictures.

**Fig. 7:**
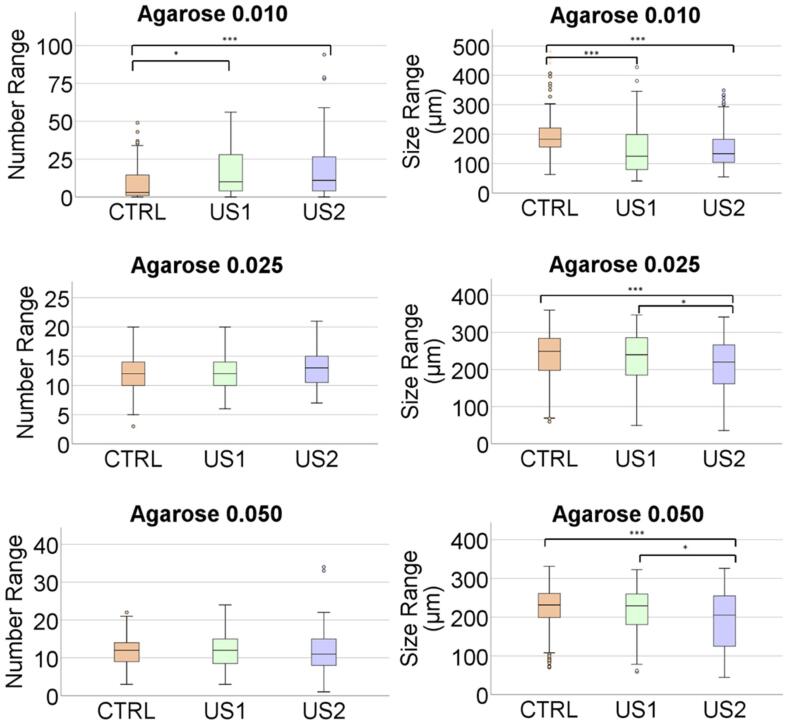
Represents the crystal numbers distribution and average size range as a function of agarose concentration. There are significant differences in all groups irradiated.

Sonocrystallyzation studies were evaluated at different agarose gel concentrations from 0.010 %, to 0.200 % (w/v) by quantifying the number of crystals and crystal size 24 h after the application of the US irradiation protocols (US1 & US2) (Fig. 6, Fig. 7). At the lowest agarose concentration (0.010 % w/v) results showed a significant increase in the number of crystals and a consequent reduction of crystals size independently of the applied protocol, although in US2 crystals size distribution was narrower (Fig. 7 and Figure S5). At agarose concentration of 0.025 % and 0.050 % (w/v) the number of crystals is similar but they show a statistically significant smaller sizes (Fig. 7) and narrower size distribution (Figure S5) than in solution. At 0.100 % and 0.200 % (w/v) agarose concentration the effect in the number of crystals and crystal sizes are not significantly different from the silent conditions (Control) meaning that the induction effect of agarose overcome the potential effect of US. This can be clearly observed in the number of crystals that are higher and of smaller size. (Figure S5 and S6).

Taking into account size distribution (Figure S5) it was clear that both experimental set-ups (US1 and US2) evolved similarly as the agarose concentration increased and became almost identical to the silent control experiments when agarose concentration was 0.100 % (w/v).

## 4 Conclusions

Sonocrystallization although very promising in protein crystallization has not been studied in-depth and contradictory results have been reported [25]. To shed light over this field and validated previous works, we have compared the effect of US in lysozyme crystallization in solution and in gel media, both experiments carried out under the same US conditions.

We have observed an induction effect of an US irradiation on the nucleation of lysozyme when crystallized in the absence or the presence of agarose at a concentration below 0.100 % w/v. In both media the effect of ultrasound is similar, the induction of the nucleation giving rise to a higher number of crystals of smaller size. Above 0.100 % w/v agarose concentration the effect of US is hindered maybe due to the enhancement of the mechanical properties of the gel.

The results obtained in solution and in agarose gels are statistically significant and corroborates the effect of US in crystallization. We have also demonstrated that the combination of both techniques, the use of hydrogel and US, are compatible in protein crystallization.

## CRediT authorship contribution statement

**Mariia Savchenko:** Formal analysis, Investigation, Validation. **Manuel Hurtado:** Formal analysis, Investigation, Validation. **Modesto T. Lopez-Lopez:** Methodology, Funding acquisition, Formal analysis, Writing – original draft. **Guillermo Rus:** . **Luis Álvarez de Cienfuegos:** Conceptualization, Funding acquisition, Methodology, Project administration, Supervision, Writing – original draft, Writing – review & editing. **Juan Melchor:** Funding acquisition, Methodology, Project administration, Supervision, Writing – original draft, Writing – review & editing. **José A. Gavira:** Conceptualization, Funding acquisition, Methodology, Project administration, Supervision, Writing – original draft, Writing – review & editing.

## Declaration of Competing Interest

The authors declare that they have no known competing financial interests or personal relationships that could have appeared to influence the work reported in this paper.
