# Supplementary material for: Lysozyme crystallization in hydrogel media under ultrasound irradiation
Source: Ultrason Sonochem. 2022 Jul 18;88:106096. doi: 10.1016/j.ultsonch.2022.106096 (PMC9305616; doi:10.1016/j.ultsonch.2022.106096)
Supplement: Supplementary data 1 [file mmc1.docx]

**Supplementary material**

to

**Lysozyme crystallization in hydrogel media under ultrasound irradiation**

Mariia Savchenko,^a,c,d^ Manuel Hurtado,^b,e,f,g^ Modesto T. Lopez-Lopez,^c,g^ Guillermo Rus,^e,f,g^ Luis Álvarez de Cienfuegos,^a,g,^* Juan Melchor,^b,f,g,^* and José A. Gavira^d,^*

^a^ Universidad de Granada (UGR), Departamento de Química Orgánica, Unidad de Excelencia Química Aplicada a Biomedicina y Medioambiente (UEQ), C. U. Fuentenueva, Avda. Severo Ochoa s/n, E-18071 Granada, Spain.

^b^ Universidad de Granada (UGR), Departamento de Estadística e Investigación Operativa, Spain.

^c^ Universidad de Granada (UGR), Departamento de Física Aplicada, C. U. Fuentenueva, Avda. Severo Ochoa s/n, E-18071 Granada, Spain.

^d^ Laboratorio de Estudios Cristalográficos, Instituto Andaluz de Ciencias de la Tierra (Consejo Superior de Investigaciones Científicas-UGR), UEQ, Avenida de las Palmeras 4, 18100 Armilla, Granada, Spain.

^e^ Departamento de Mecánica de Estructuras e Ingeniería Hidráulica, Universidad de Granada, Spain, Ultrasonics Lab TEP-959, Universidad de Granada, Spain

^f^ Unidad de Excelencia Modeling Nature MNAT, Universidad de Granada, Spain

^g^ Instituto de Investigación Biosanitaria Ibs. GRANADA, Granada, Spain.

| 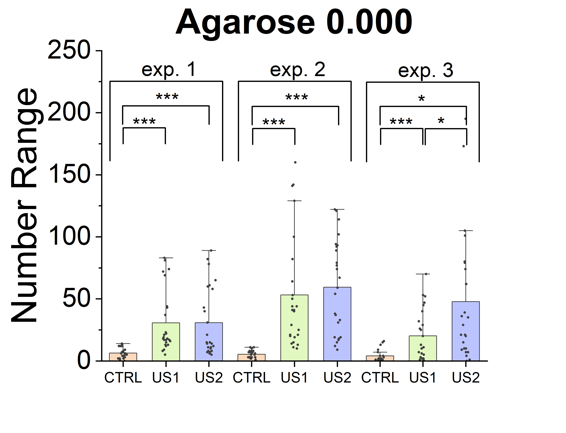 | 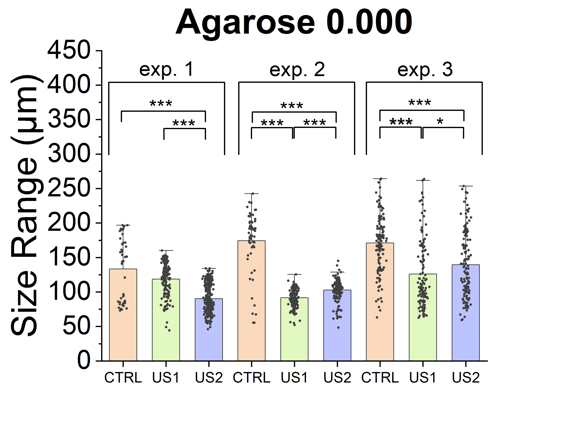 |
| --- | --- |
| 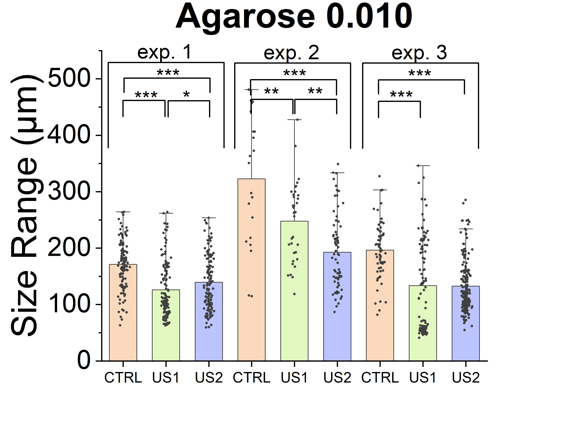 | 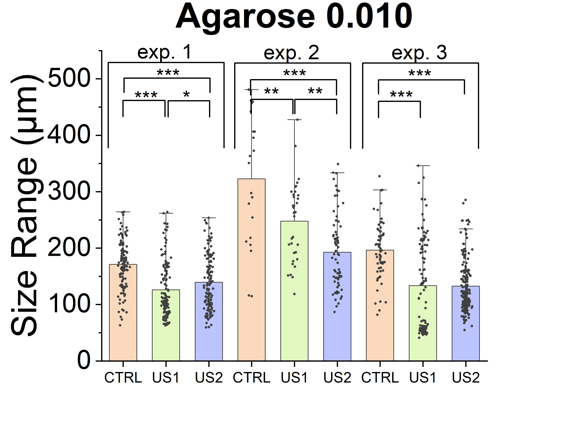 |
| 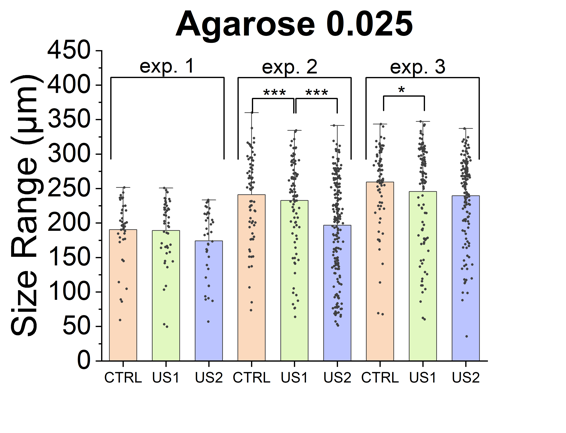 | 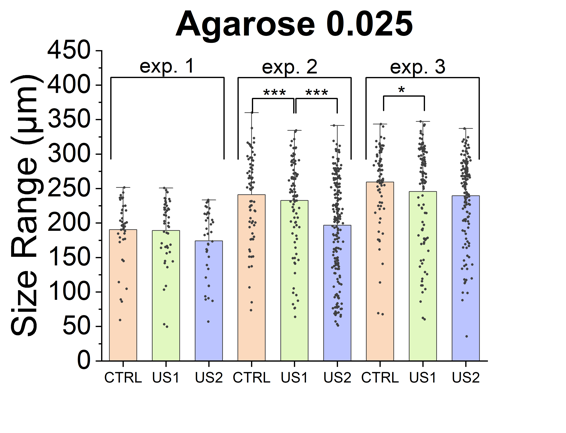 |
| 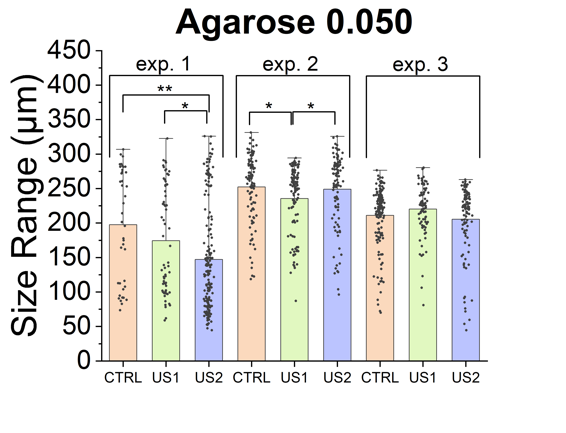 | 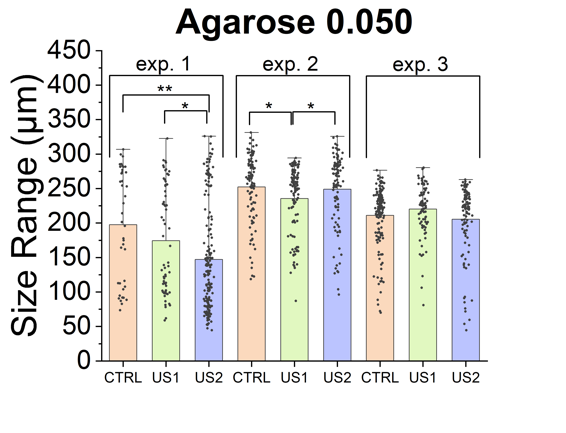 |

**Figure S1.** Statistical analysis of the number (left column) and size (right column) of crystals grown in solution and in agarose gel at different concentration applying protocols US1 and US2.

| 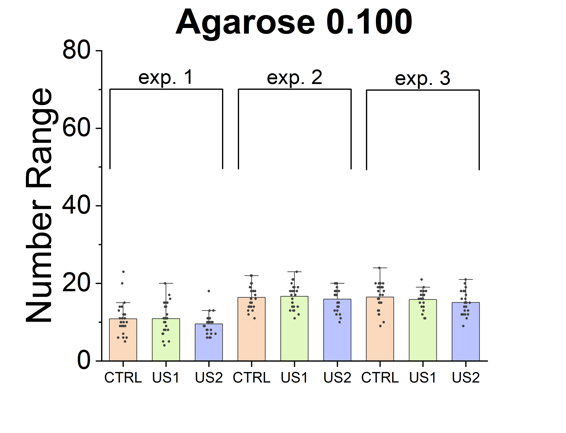 | 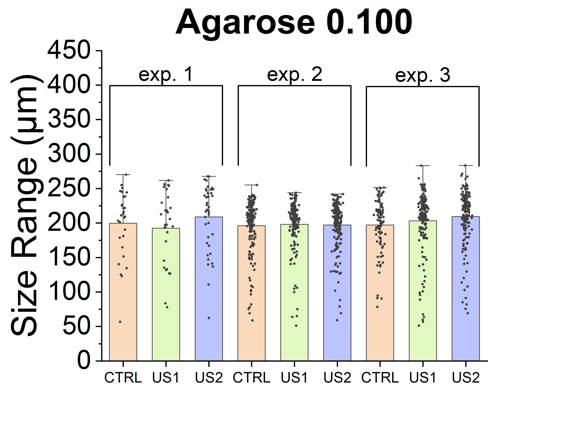 |
| --- | --- |
| 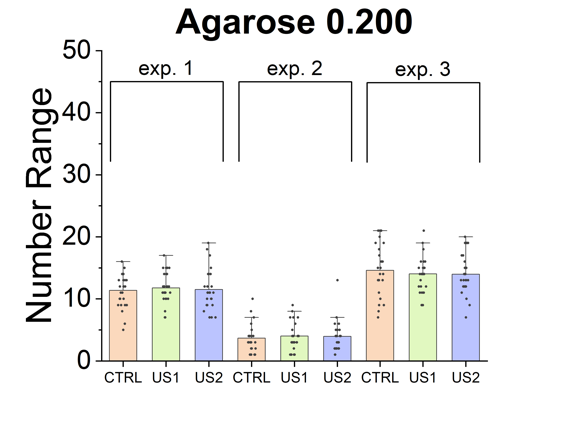 | 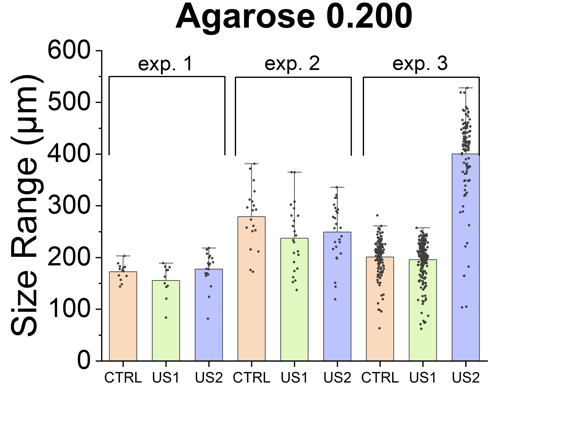 |

**Figure S2.** Statistical analysis of the number (left column) and size (right column) of crystals grown in agarose gel at different concentration applying protocols US1 and US2.

**Figure S3.** Distribution of lysozyme crystals size obtained in solution under silent (control) and ultrasonic (US1 & US2) crystallization conditions for the three experiments.


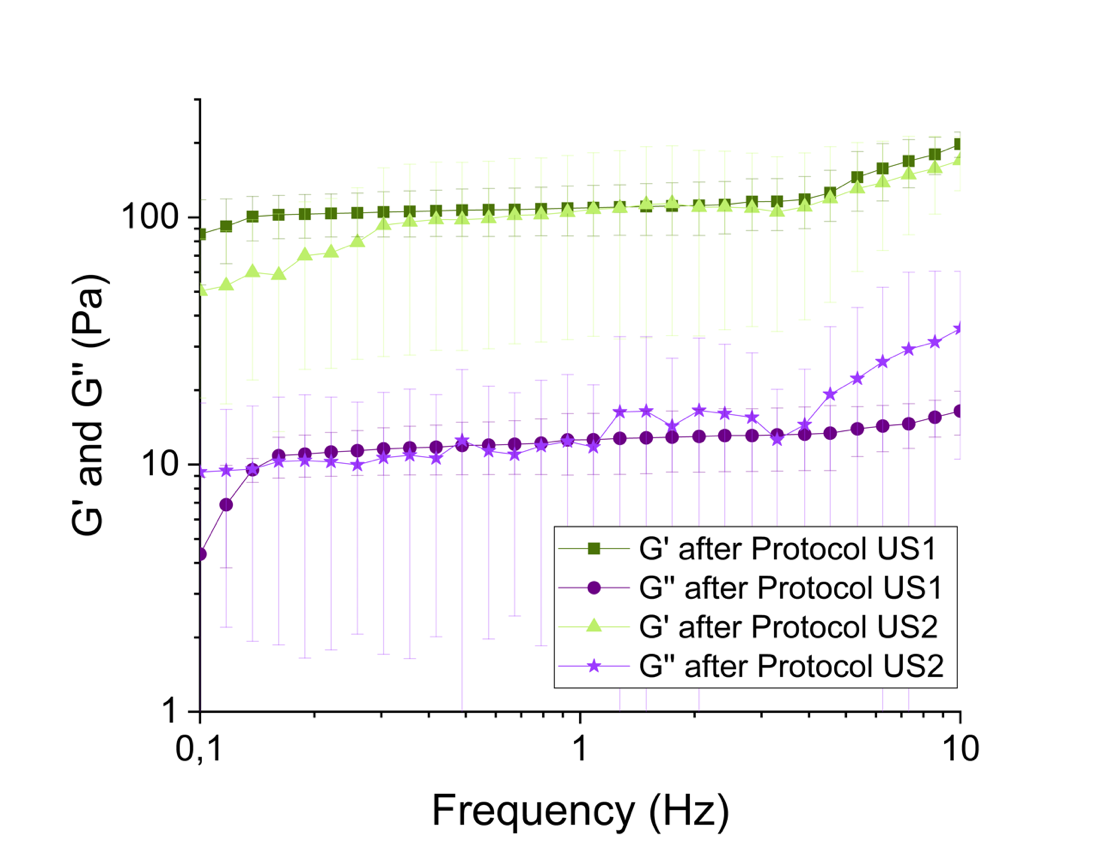


**Figure S4.** Viscoelastic moduli as a function of frequency at a constant stress of 1 Pa for 0.200% agarose gels after applying the protocol US1 and US2. For each point (mean value) we represent the standard deviations. Note that the nonsymmetric appearance of standard deviations is due to the logarithmic scale.


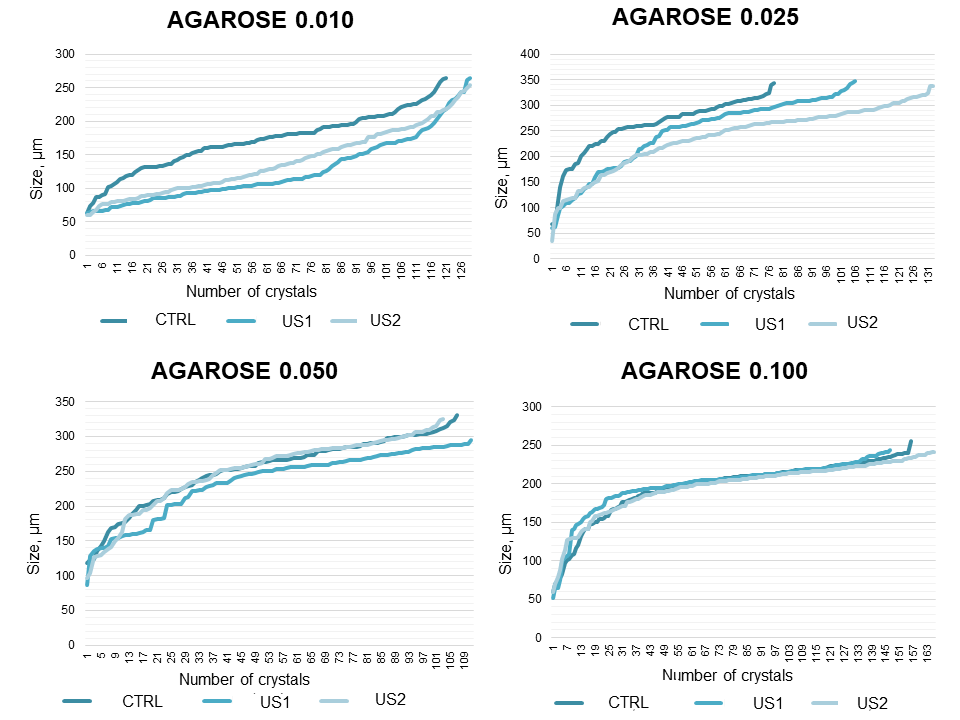


**Figure S5.** Example of the istribution of lysozyme crystals size obtained at different agarose concentration from 0.01% to 0.1 % (w/v) silent or under the influence of ultrasonic pulse (protocols US1 & US2).

**Figure S6.** Lysozyme crystals obtained in solution under silent condition (Control) and ultrasonic irradiation for 30 minutes immediately after preparing the experiment (US1) and 30 minutes after preparation (US2). The scale bar in optical microscopy images is 500 µm in all the pictures.
